# NAC Transcription Factor PwNAC11 Activates *ERD1* by Interaction with ABF3 and DREB2A to Enhance Drought Tolerance in Transgenic *Arabidopsis*

**DOI:** 10.3390/ijms22136952

**Published:** 2021-06-28

**Authors:** Mingxin Yu, Junling Liu, Bingshuai Du, Mengjuan Zhang, Aibin Wang, Lingyun Zhang

**Affiliations:** Key Laboratory of Forest Silviculture and Conservation of the Ministry of Education, The College of Forestry, Beijing Forestry University, Beijing 100083, China; ymxbjfu@163.com (M.Y.); liujunling022@163.com (J.L.); dubingshuai624@163.com (B.D.); liujunling02@126.com (M.Z.); wangaibin126@126.com (A.W.)

**Keywords:** *Picea wilsonii*, transcription factor, PwNAC11, drought stress, ABA signaling

## Abstract

NAC (NAM, ATAF1/2, and CUC2) transcription factors are ubiquitously distributed in eukaryotes and play significant roles in stress response. However, the functional verifications of NACs in *Picea (P.) wilsonii* remain largely uncharacterized. Here, we identified the NAC transcription factor PwNAC11 as a mediator of drought stress, which was significantly upregulated in *P. wilsonii* under drought and abscisic acid (ABA) treatments. Yeast two-hybrid assays showed that both the full length and C-terminal of PwNAC11 had transcriptional activation activity and PwNAC11 protein cannot form a homodimer by itself. Subcellular observation demonstrated that PwNAC11 protein was located in nucleus. The overexpression of *PwNAC11* in *Arabidopsis* obviously improved the tolerance to drought stress but delayed flowering time under nonstress conditions. The steady-state level of antioxidant enzymes’ activities and light energy conversion efficiency were significantly increased in PwNAC11 transgenic lines under dehydration compared to wild plants. *PwNAC11* transgenic lines showed hypersensitivity to ABA and PwNAC11 activated the expression of the downstream gene *ERD1* by binding to ABA-responsive elements (ABREs) instead of drought-responsive elements (DREs). Genetic evidence demonstrated that PwNAC11 physically interacted with an ABA-induced protein—ABRE Binding Factor3 (ABF3)—and promoted the activation of *ERD1* promoter, which implied an ABA-dependent signaling cascade controlled by PwNAC11. In addition, qRT-PCR and yeast assays showed that an ABA-independent gene—DREB2A—was also probably involved in PwNAC11-mediated drought stress response. Taken together, our results provide the evidence that PwNAC11 plays a dominant role in plants positively responding to early drought stress and ABF3 and DREB2A synergistically regulate the expression of *ERD1*.

## 1. Introduction

Water deficit is one of the most disruptive abiotic stresses influencing plant growth and development [1,2]. As environments of plants have gradually deteriorated as a result of global warming, it is of great significance to understand the molecular mechanisms of plants themselves responding to abiotic stress, especially drought stress, for further cultivating new varieties with strong adaptability. In order to fight against abiotic stress, plants have evolved a series of sophisticated but effective strategies to protect themselves from the environmental disadvantages, including stress escape and stress tolerance [3,4]. Stress escape mainly occurs under mild stress conditions, which allows plants to accelerate the growth and flowering processes in order to avoid prematurely dying of water deprivation. Stress tolerance is a regulatory mechanism by which plants can endure long-term stress and maintain their vitality under such environmental pressure [5].

Particularly, transcription factors (TFs) play a vital role in stress regulatory mechanisms by selectively binding to *cis*-elements in the promoter of downstream genes [6]. NACs are one of the most widely reported families of transcription factors in plants, which can be divided into NAM, ATAF and CUC subfamilies according to their interspecific DNA-binding domains. The N-terminal domain of NAC TF can be divided into five conserved subdomains (A, B, C, D, and E). Among them, the A domain is related to the formation of dimers, the C and D domains are highly conserved with binding affinity to promoters, but the B and E domains are relatively non-conserved. By comparison, the C-terminal exhibits more structural variability and is considered as the transcription activation domain [7]. Ooka et al. conducted a comprehensive analysis of 180 NAC family proteins in *Arabidopsis* and rice for the first time [8]. According to the similarity of the NAC domain sequences between *Arabidopsis* and rice, the 180 proteins were divided into two large groups and 18 subgroups. A growing body of evidence has shown that NAC transcription factors are widely involved in plant response to abiotic/biotic stress [9], senescence [10], fruit ripening [11] and hormonal regulation [12], etc.

Over the years, a variety of NAC TFs in different plant species have been reported to play positive roles in response to adverse environments. For example, three NAC transcription factors—ANAC019, ANAC055 and ANAC072—were substantially induced by drought, salt and ABA treatments, the overexpression of which improved the drought tolerance of transgenic *Arabidopsis* [13]. Similarly, the overexpression of pepper *CaNAC46* in *Arabidopsis* significantly inhibited ROS accumulation and the transgenic lines are less susceptible to salt stress [14]. Similarly, *TaSNAC8-6A* overexpression in wheat exhibited increased drought stress tolerance and this process was proven to be controlled by the auxin- and drought response pathways by transcriptomic analysis [15]. The NAC family member JUB1 was proven to be a regulator of drought stress, and the overexpression of tomato *SlJUB1* or *Arabidopsis AtJUB1* could increase drought tolerance in transgenic tomato by directly binding to the promoters of *SlDELLA*, *SlDREB2* and *SlDREB1* [16], suggesting that the regulation mechanisms of the NAC transcription factors involved in abiotic stress are relatively specific among different species. In woody plants, BpNAC012 derived from *Betula platyphylla* activated the core CGTG/A elements to promote the expression of stress-responsive genes, which further enhanced osmotic and salt stress tolerance in *BpNAC012* transgenic birch [17]. In addition, many of the NAC TFs were demonstrated to regulate abscisic acid (ABA)-induced stress response process. *ONAC022* showed transcriptional abundance after ABA and drought treatments in rice and *NAC022* OE lines improved sensitivity to exogenous ABA [18]. ANAC096 could directly interact with ABF2 and ABF4 to activate the expression of *RD29A*, thus participating in dehydration and osmotic stresses regulation in an ABA-dependent pathway in *Arabidopsis* [19]. Although the functions of the NAC TF family have been investigated in various plant species, it has been difficulties of in-depth study for revealing the complicated and efficient molecular mechanisms inside.

ABA, an important hormone upstream of transcription factors, acts as a messenger in the biological processes of stomatal conductance, seed dormancy and germination, leaf senescence, and stress response [20,21,22]. Once suffering biotic or abiotic stress, the content of ABA shows a notable increase, and endogenous ABA will be transported to the ground part through the transmission of xylem to reduce stomatal conductance, further reducing water loss and activating the corresponding receptors to initiate signal transduction in response to stress [23]. ABA synthesis in plants is mainly formed through the degradation of carotenoids: several crucial enzymatic proteins, such as NCED, ABA2 and AAO, are involved in this process [24]. The transduction of the ABA signal mainly consists of three key ABA receptors, PP2C, PYP/PYL/RCAR, and SNRK2 kinase [25]. In the presence of ABA, SnRK2 could phosphorylate downstream ABA-responsive element (ABRE)-binding factors (ABFs). Meanwhile, some transcription factors such as NAC, bZIP, MYB and WRKY, are reported to activate the expression of ABA-dependent genes through binding to cis-acting elements on promoters of downstream genes containing specific ABRE motifs. In addition, other cis-acting regulatory elements, such as dehydration responsive elements (DREs) are also demonstrated to respond to external stresses in an ABA-independent pathway [26,27].

Although the roles of NAC transcription factors involved in abiotic stress response have been extensively explored, the knowledge regarding NAC TFs participating in the regulation of coniferous forests responding to abiotic stress is still limited. *Picea wilsonii*, an endemic species of coniferous forests, which is best characterized by tolerance to abiotic stress and environment adaptability, is widespread in northern China. *Picea wilsonii* also has ornamental value in landscaping based on its impressing appearance and lush green crown [28]. Given its advantageous characteristics, it is of great significance to screen and reveal the molecular mechanisms of its abiotic resistance. Previously, we identified two NAC transcription factors—PwNAC2 and PwNAC30—from *P. wilsonii*, which acted as positive and negative regulators in response to abiotic stress, respectively [28,29], suggesting the complexity and functional diversity of NAC TF members in coniferous forests. Here, based on the high-throughput RNA-sequencing (RNA-seq) of the expression profiles of *P. wilsonii* in the absence or presence of drought treatments [30], we identify PwNAC11 as one of the most differentially expressed genes (DEGs) on a transcriptional level and further elucidate the function and molecular mechanism underlying the ABA-mediated drought stress response. We found that overexpression of *PwNAC11* in *Arabidopsis* obviously improved tolerance to drought stress by binding to the promoter of *ERD1*. During this process, the interaction of PwNAC11 with ABF3 synergistically activated the expression of the *ERD1* promoter, resulting in increased resistance to drought stress. The biochemical and physiological evidence showed that ABA positively promoted the regulation of the PwNAC11-mediated abiotic stress response. Our study provides the basis for a new understanding of NAC TF in genetic breeding and the improvement of coniferous forest germplasm resources.

## 2. Results

### 2.1. Bioinformatics Analysis of PwNAC11

**Figure 1:**
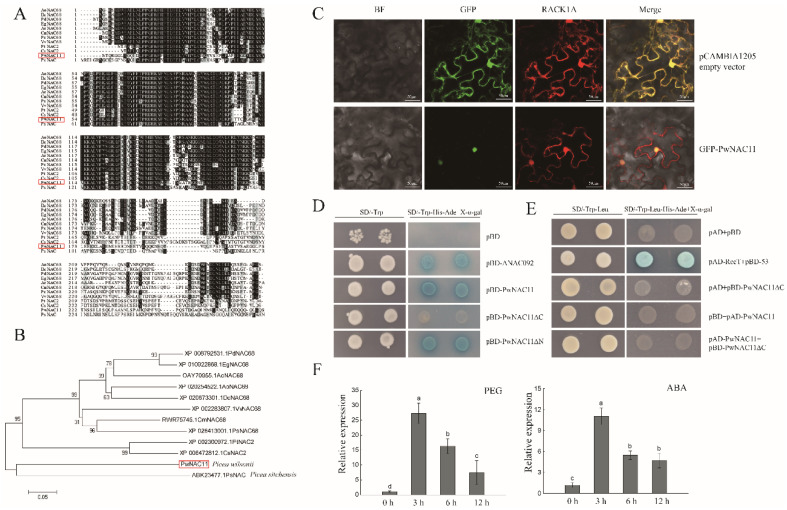
(**A**) Protein multiple sequence alignment of PwNAC11. (**B**) Phylogenetic analysis of PwNAC11. Numbers on each branch represent the confidence of 1000 repetitions. (**C**) Subcellular localization of PwNAC11. RACK1A-RFP is used as a marker gene which locates in nucleus, cytoplasm and cell membrane. The bar in each picture is 10 μm. (**D**) Yeast trans-acting activity assay. pBD-ANAC092 was used as positive control. pBD-PwNAC11ΔN refers to the PwNAC11 protein without N terminal (171–318) and pBD-PwNAC11ΔC refers to the PwNAC11 protein without C terminal (1–153). (**E**) Yeast two-hybrid assay for PwNAC11 forming homodimers. pAD-RecT+pBD-53 was used as positive control and pAD + pBD was used for negative control. (**F**) Expression profiles of *PwNAC11* in *P. wilsonii* under drought and ABA treatments. Different letters indicate the significant differences at *p* < 0.05.

*PwNAC11* was cloned from cDNA library of *P. wilsonii*. It has an open reading frame with a length of 954 bp and encodes a protein with 317 amino acid. Protein multiple sequence alignment and BLAST analysis showed that the PwNAC11 protein had a typical NAM (no apical meristem) domain without transmembrane domain (TMD) (Figure 1A). Phylogenetic analysis indicated that PwNAC11 was close to the homology of *Picea sitchensis* (Figure 1B).

To determine whether PwNAC11 potentially functions as a transcription factor, we first examined the subcellular localization of the PwNAC11 protein. The results showed that the fluorescent signals for the empty vector were widely detected in the nucleus, cell membrane, and cytoplasm, but the PwNAC11-GFP fusion protein was only detected in the nucleus, indicating that PwNAC11 is a nucleus-located transcription factor (Figure 1C). The yeast two-hybrid assay was conducted to detect whether PwNAC11 had transcriptional activating activity. We found that only the yeast cells containing pGBKT7-PwNAC11, pGBKT7-PwNAC11-C, and pGBKT7-ANAC092 grew well on SD/Trp-His-Ade selective medium, suggesting that both full length and C-terminal of PwNAC11 have transcriptional activating activity (Figure 1D). Moreover, yeast strains AH109 co-transformed with pGBKT7-PwNAC11 + pGADT7-PwNAC11 and pGBKT7-PwNAC11ΔC + pGADT7-PwNAC11 cannot grow on SD/Trp-Leu-His-Ade selective medium, indicating that PwNAC11 cannot form a homodimer by itself (Figure 1E).

To elucidate the response of PwNAC11 to drought stress, qRT-PCR was performed with 4-week-old *P. wilsonii* seedlings. The results showed that the expression of *PwNAC11* was dramatically induced at 3 h by PEG or ABA treatment, indicating that PwNAC11 is probably involved in the process of plants’ early responses to adverse environments (Figure 1F).

### 2.2. Overexpression of PwNAC11 Enhances Drought Tolerance in Transgenic A. thaliana

**Figure 2:**
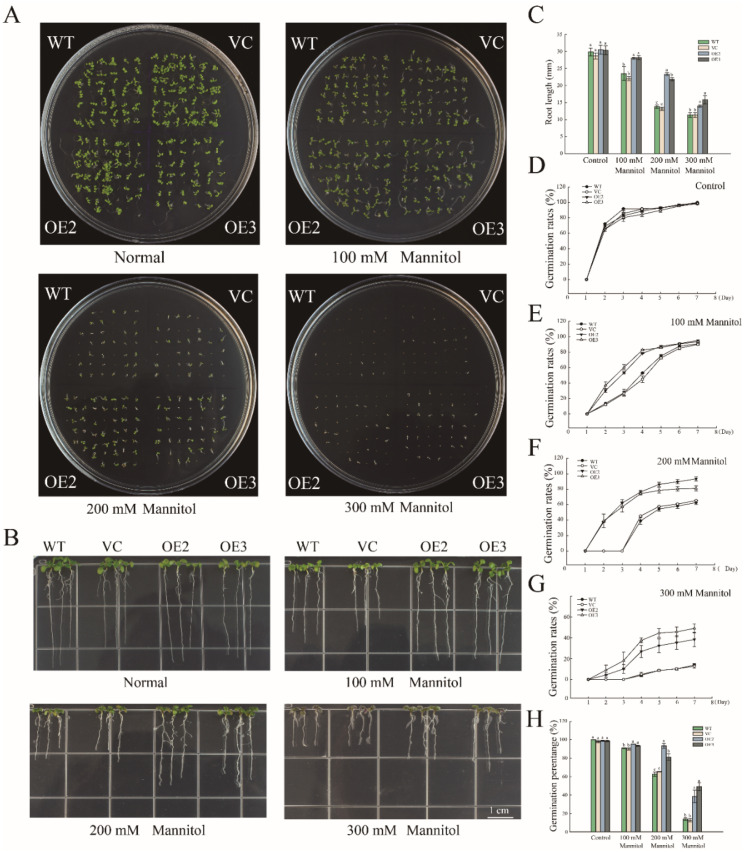
PwNAC11 overexpression promotes seed germination under simulated drought conditions. (**A**) Germination assay under different concentrations of mannitol treatments. (**B**) Root elongation assays under different concentrations of mannitol treatments. (**C**) Quantification of root length under different concentrations of mannitol treatments. Different letters indicate significant differences at *p*-value < 0.05. (**D**–**G**) Germination rates of different lines under different concentrations of mannitol treatments within 7 days. (**H**) Germination percentage of each line after 7-day simulated drought treatment. Different letters indicate significant differences at *p*-value < 0.05.

In order to investigate the function of PwNAC11 in response to drought stress, we heterogeneously expressed *PwNAC11* in *Arabidopsis* under the control of CaMV 35S promoter. Two T3 lines with *PwNAC11* highly expressed, named OE2 and OE3, were selected for further functional analysis (Figure S1). The seeds of wild type (WT), empty vector (VC) and overexpression (OE2 and OE3) lines were sown on MS medium with selected concentrations of mannitol. It was found that there was no significant difference in the germination rates among these lines under normal conditions. Under the simulated drought conditions, the growth of the WT and VC groups were severely inhibited, whereas the germination rates of the seeds of the two OE lines were less affected (Figure 2A). For example, the germination percentage of OE lines were over 80% under mannitol conditions of 200 mM compared to less than 60% of the WT and VC lines (Figure 2F,H). Moreover, the *PwNAC11* transgenic lines showed stronger germination rates. Under 100 mM mannitol treatment, the germination rates of OE lines were 1.6 times higher than control groups on the 4th day. Meanwhile, in the presence of 300 mM mannitol, the germination rate of OE3 line was nearly 20% on the 3rd day, but the seeds of control groups showed almost no germination at the same time, implying that the OE lines had stronger seed vitality at the early stage of germination under drought treatment. Similar results were found in the root length experiment, especially under the treatment of 200 mM mannitol, in which the average root length of the OE lines was 10 mm longer than that of WT and VC groups (Figure 2B,C).

**Figure 3:**
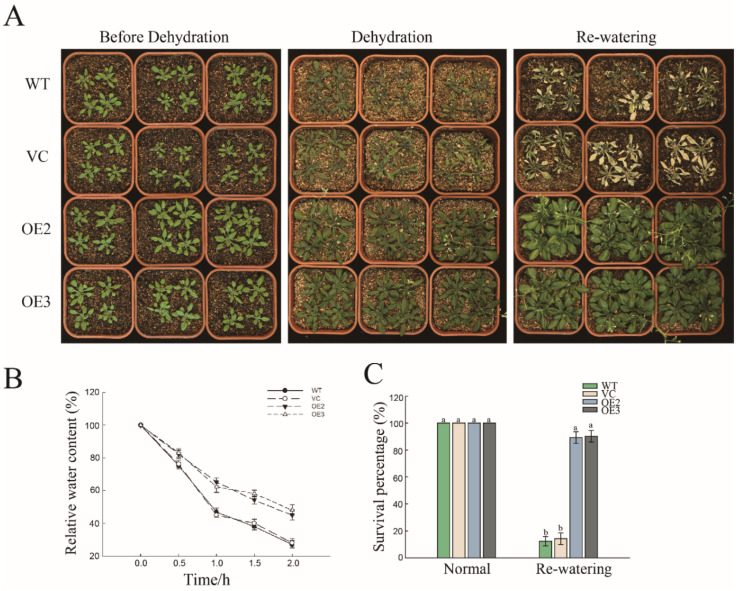
Overexpression of *PwNAC11* improves tolerance to drought stress. (**A**) Phenotypes of different lines under drought conditions. Dehydration lasted 14 days, followed by re-watering for 3 days. (**B**) Relative Water Content (RWC) after drought stress. Isolated leaves were placed at room temperature and dehydrated for 2 h. (**C**) Survival percentage before drought treatment and after re-watering. Different letters indicate the significant differences at *p* < 0.05.

To analyze the role of PwNAC11 in drought stress during adult stage, 4-week-old seedlings were exposed to drought treatment. We found that the transgenic lines only wilted slightly and still maintained a normal growth state with a final survival percentage of 74% after drought treatment and followed by re-watering (Figure 3C), but the leaves of most of the WT and VC plants were wilted seriously or even dead, which were unable to recover after re-watering (Figure 3A). We also counted the relative water content (RWC) of each line. The RWC of the OE lines was approximately 50%, while it was only 25% for WT and VC lines (Figure 3B).

### 2.3. PwNAC11 Is Involved in the Regulation of ROS Accumulation and Photosynthetic Efficiency under Drought Conditions

**Figure 4:**
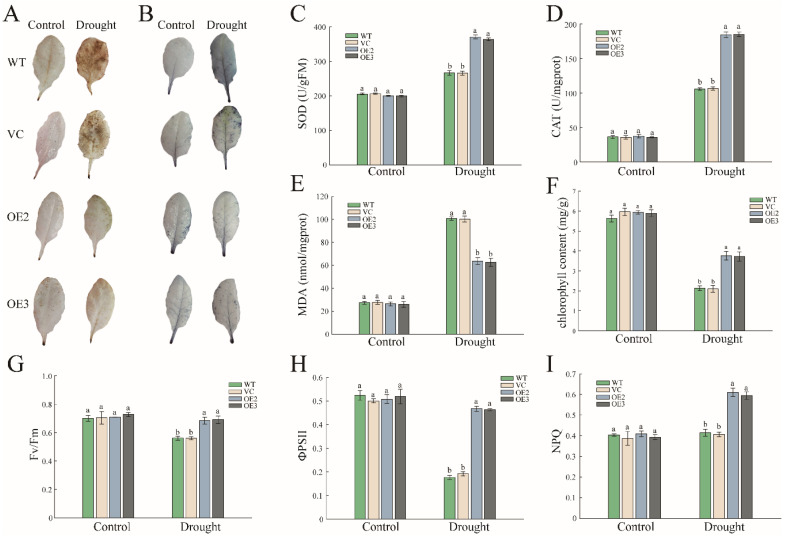
Overexpression of PwNAC11 enhances ROS scavenging ability in transgenic *Arabidopsis*. (**A**) Histochemical detection of hydrogen peroxide using DAB staining. (**B**) Histochemical detection of superoxide using NBT staining. (**C**) The activity of SOD. (**D**) The activity of CAT. (**E**) The content of MDA. (**F**) The content of chlorophyll. (**G**) Measurement of Fv/Fm under normal and drought conditions. (**H**) Measurement of ΦPSII under normal and drought conditions. (**I**) Measurement of NPQ under normal and drought conditions. Different letters indicate significant differences at *p*-value < 0.05.

Drought stress has been reported to accelerate the accumulation of ROS. In order to investigate whether PwNAC11 promotes the degradation of superfluous hydrogen peroxide and superoxide in the leaves, we conducted the DAB and NBT staining. It was found that the leaves of each line were hardly stained under normal conditions. Nevertheless, once submitted to drought stress, the staining intensity of the leaves for WT and VC groups was substantially deeper than those of OE lines (Figure 4A,B), suggesting more ROS accumulated in WT and VC groups compared with OE lines. Further antioxidant enzymes activity assay indicated the activities of SOD and CAT increased apparently in two OE lines during drought treatments compared to normal conditions (Figure 4C,D). The severity of lipid oxidation was reflected in the MDA content. Upon drought treatment, the content of MDA in the OE lines was below 80 nmol/mg, which was 37% lower than that in WT and VC lines (Figure 4E). These results indicate that overexpression of *PwNAC11* significantly improved the scavenging abilities of ROS and contributed to the protection from peroxidation damage under drought stress. In addition, the decomposition rate of chlorophyll was reduced in transgenic lines (Figure 4F), suggesting that PwNAC11 probably participates in the regulation of the photosynthetic system.

Drought stress may also reduce the photosynthetic rate, leading to the decline in the efficiency of photosynthesis (Fv/Fm) and quantum yield of PSII electron transport (ΦPSII). In order to verify whether PwNAC11 is involved in the protection of plant photosynthesis under drought stress, several photosynthetic parameters were subsequently measured after 14 days of drought stress. It was observed that Fv/Fm fell by 22% in WT and VC after exposure to drought stress, but it was almost unchanged in the two OE lines (Figure 4G). Similarly, the ΦPSII decreased significantly by 65.4% in WT and only by 10% in OE lines (Figure 4H). On the contrary, nonphotochemical quenching (NPQ) increased dramatically in the OE lines under drought treatment and showed no significant difference in the WT and VC groups in the absence or presence of drought treatment (Figure 4I), suggesting that PwNAC11 confers increased photoprotection in OE lines.

### 2.4. Overexpression of PwNAC11 Activates the Expression of Stress-Responsive Genes

**Figure 5:**
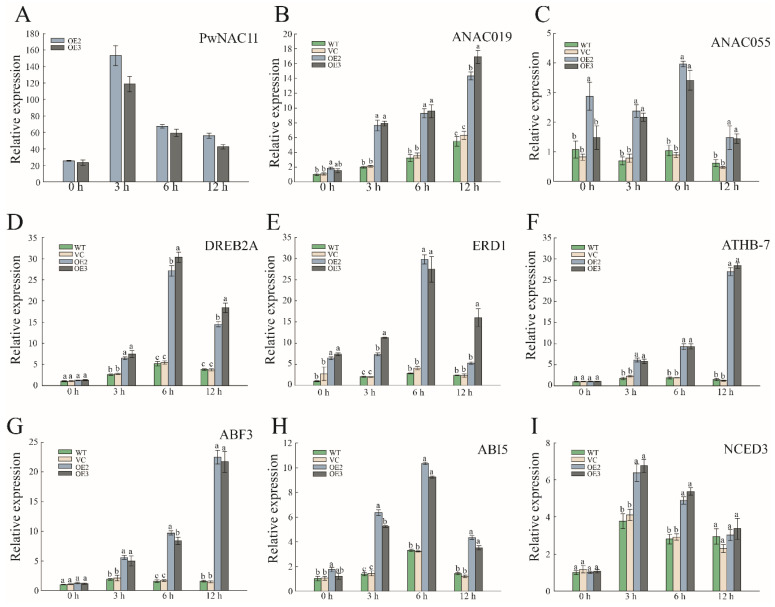
Expression profiles of some stress-responsive genes and ABA-responsive genes after PEG treatment. (**A**–**I**) Expression profiles of these genes in OE, WT, and VC lines under simulated drought conditions. The data are represented as means ± standard error from three independent replications. Different letters indicate significant differences at *p*-value < 0.05.

Our study found that overexpression of *PwNAC11* improved plant resistance to drought stress and can be greatly induced by ABA. To further quantify the expression levels of stress-responsive and ABA-induced genes of different lines, we selected such genes as *ANAC019*, *ANAC055*, *DREB2A*, *ERD1*, *ATHB-7* and *ABF3* for qRT-PCR analysis based on the reports on the regulation network of homologous genes of *PwNAC11* in *Arabidopsis* (Figure 5). Under simulated drought conditions, most of these genes showed higher expression levels in OE lines compared with the WT and VC groups. *ANAC055* and *ERD1* were even upregulated in normal conditions compared with WT and VC groups (Figure 5C,E). For *ERD1*, the expression was 12 times higher in OE lines than that in the other groups under PEG treatment, and *ATHB-7* showed more than a twenty-fold increase when subjected to 12 h PEG treatment (Figure 5F). These results suggested that PwNAC11 promoted the expression of stress-related and ABA-responsive genes, and thus, improved the stress tolerance in the plants.

### 2.5. Overexpression of PwNAC11 Increases ABA Sensitivity and Promotes ABA-Induced Stomatal Closure in A. thaliana

**Figure 6:**
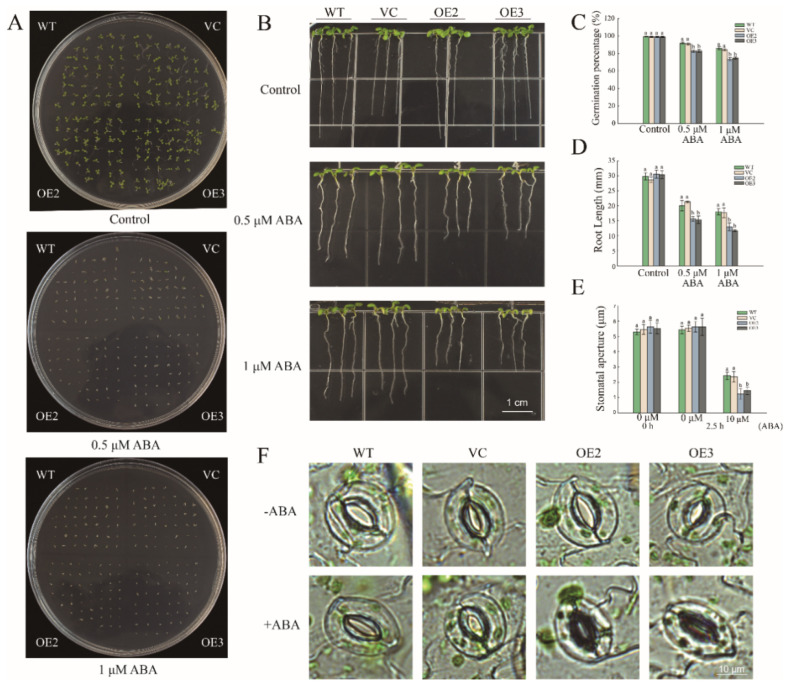
The stomatal aperture of *PwNAC11* overexpression lines was positively modulated by ABA. (**A**) Germination assays for different lines under different concentrations of ABA treatments. (**B**) Root length assays for different lines under different concentrations of ABA treatments. (**C**) Quantification of germination percentage under ABA treatments. (**D**) Quantification of root length under ABA treatments. (**E**) Quantitative comparisons of stomatal aperture calculated by ImageJ software. (**F**) The observation of stomatal aperture of OE, WT and VC lines photographed by biomicroscope (DM2500, Leica). Different letters indicate the significant differences at *p* < 0.05.

Since the expression of *PwNAC11* was upregulated under exogenous ABA treatment in *P. wilsonii*, we speculate that PwNAC11 is responsive to the ABA signal. To prove this hypothesis, we conducted another phenotypic experiment by exposing the seeds of each line to different concentrations of exogenous ABA to test the response of PwNAC11 to the ABA signal. We found that the germination percentage of OE2 and OE3 decreased to 70% under 1 μM ABA treatment, while it was maintained at nearly 80% in the WT and VC lines (Figure 6A,C). The results of the root length experiment also proved that the root length of the OE lines was almost 5 mm shorter than that of the WT and VC lines, whether under 0.5 μM or 1 μM ABA treatment (Figure 6B,D).

Recent studies indicated that increased ABA content could promote stomatal closure to improve the drought tolerance of plants [31]. We further observed and calculated the level of close stomata by exogenously applying ABA in different lines. The results showed that there was no distinct discrepancy under normal light conditions. Once adding 10 μM ABA, however, an acceleration of stomatal closure occurred in all plants, of which the OE lines exhibited a more obvious response to exogenous ABA treatment (Figure 6E,F). These results indicated that *PwNAC11* overexpression lines were more sensitive to exogenous ABA.

### 2.6. PwNAC11 Interacts with ABF3 and DREB2A

**Figure 7:**
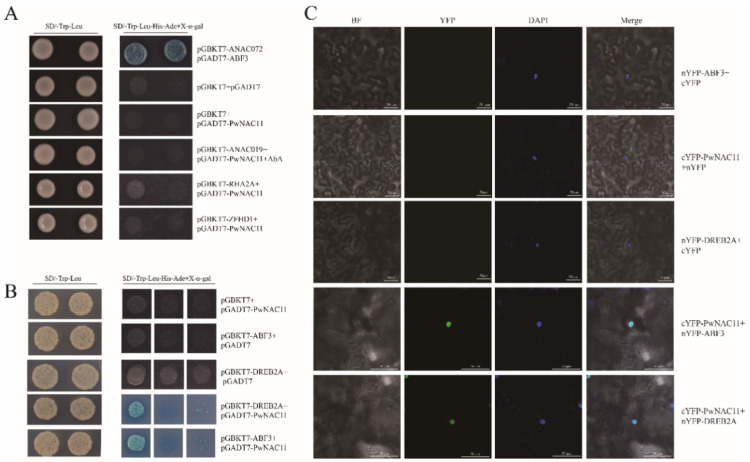
PwNAC11 interacts with ABF3 and DREB2A. (**A**) Yeast two-hybrid assays for PwNAC11 with ANAC019, RHA2A and ZFHD1. The yeast strains containing pGADT7-ABF3 and pGBKT7-ANAC072 fusion plasmids were used as positive control. Empty pGADT7 and pGBKT7 were used as negative control. (**B**) Yeast two-hybrid assays for PwNAC11 with ABF3 and DREB2A. (**C**) BiFC assays for PwNAC11 and ABF3/DREB2A interactions. Bars represent 50 μm. The C-terminal or N-terminal of YFP was used as negative control.

According to the prediction of the STRING and related reports of *PwNAC11* homologous genes in *Arabidopsis*, five proteins were selected for potential interaction verification (Figure 7A). The transcriptional activity results showed that only pGBKT7-ANAC019 could grow well on selective medium and activate the reporter gene expression, which implied that ANAC019 has transcriptional activity, but not for other chosen proteins (Figure S2). Subsequent yeast two-hybrid (Y2H) assays revealed that the yeast cells containing pGADT7-PwNAC11 + pGBKT7-ABF3 and pGADT7-PwNAC11 + pGBKT7-DREB2A grew well on SD/Trp-Leu-His-Ura selective medium (Figure 7B), suggesting that PwNAC11 can interact with ABF3 and DREB2A in yeast. Bimolecular fluorescence complementation (BiFC) assay further demonstrated that the YFP signals were observed in the nuclei of tobacco leaves co-expressing PwNAC11 and ABF3 or DREB2A (Figure 7C), which confirmed that PwNAC11 physically interacted with ABF3 or DREB2A in vivo.

### 2.7. PwNAC11 and ABF3 Combine with the Promoter Region of ERD1

**Figure 8:**
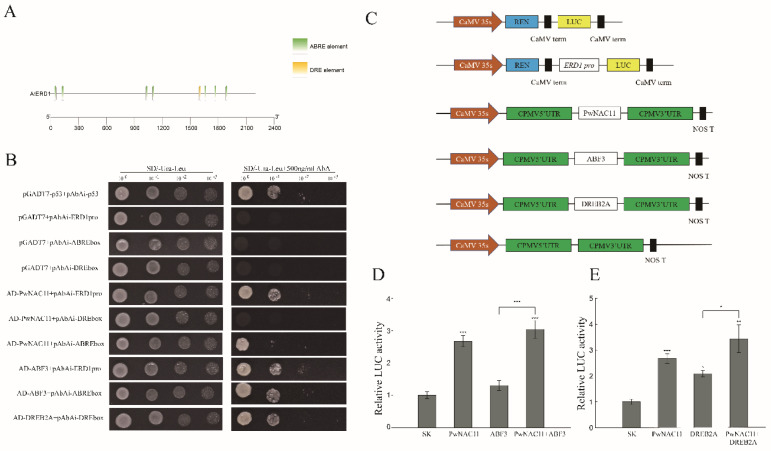
PwNAC11 and ABF3 cooperatively activates *ERD1* transcription. (**A**) Diagram of the *ERD1* promoter. The seven ABREs detected in *ERD1* promoter are indicated in green and one DRE is marked in yellow. (**B**) Yeast one-hybrid assays for the interaction of PwNAC11 with *ERD1* promoter, ABRE and DRE element. Aureobasidin A (AbA) of 500ng/mL was used to inhibit autoactivation. pGADT7-p53 and pAbAi-p53 were used as positive control. Empty PGADT7 was used as negative control. (**C**) Sketch map of the double-effector and reporter plasmids in dual-luciferase reporter assay. (**D**) Dual-luciferase assays for PwNAC11 and ABF3 interactions. (**E**) Dual-luciferase assays for PwNAC11 and DREB2A. The data are represented as means ± standard error from three independent replications. The grey lines indicate the comparisons and asterisks indicate significant differences compared with SK (* *p* < 0.05; ** *p* < 0.01; *** *p* < 0.001).

Considering that *ERD1* could be activated by *ANAC019/ANAC072* in *Arabidopsis* [32], and the expression of *ERD1* was prominently upregulated in *PwNAC11* OE lines, we subsequently conducted the yeast one-hybrid (Y1H) assays to confirm the regulation of PwNAC11 on *ERD1* promoter region. In contrast with the negative control, both pGADT7-PwNAC11 + pAbAi-*ERD1*pro and pGADT7-ABF3 + pAbAi-*ERD1*pro yeast strains could grow on SD/Ura-Leu + AbA selective medium (Figure S3), suggesting both PwNAC11 and ABF3 can combine with the *ERD1* promoter (Figure 8A,B). As ABRE and DRE motifs were enriched in the promoter region of *ERD1*, we performed another Y1H assay to test whether PwNAC11 and ABF3 could bind to the specific cis-elements of *ERD1* promoter. The results showed that both PwNAC11 and ABF3 could specifically combine with ABRE motifs, but neither PwNAC11 nor ABF3 could bind to the DRE motifs (Figure 8C). These results implied that PwNAC11 and ABF3 synergistically regulate the *ERD1* expression by binding to the ABRE motifs. Furthermore, we performed dual-luciferase assays in transformed tobacco. As is shown in Figure 8D, the expression of PwNAC11 promoted the activation of reporter gene by 2.67-fold than empty vector in tobacco, while ABF3 limitedly activated the LUC expression. The simultaneous expression of PwNAC11 and ABF3 together enhanced the activation of *ERD1* promoter than PwNAC11 alone, which were 3.04 times higher than in the control group (Figure 8D). These results proved that PwNAC11 functions as a positive regulator of the expression of *ERD1*, and co-expression of PwNAC11 and ABF3 further improves this activation by binding to the ABRE motifs. Moreover, co-expression of PwNAC11 and DREB2A also activated the expression of *ERD1* promoter (Figure 8E), suggesting that the regulation of PwNAC11 on *ERD1* was also involved in an ABA-independent signaling pathway.

### 2.8. PwNAC11 Overexpression Delays Flowering

To test whether overexpression of *PwNAC11* affected the growth and development of plants, some relative parameters were calculated. Under nonstress conditions, the rosette leaf morphology, plant height and pod length of different lines showed almost no differences (Figure S4A,B,D,E). However, the OE lines showed a 4–5-day delayed flowering phenotype compared with the WT and VC groups (Figure S4C). Meanwhile, we also examined the expression of some flowering-related genes by qRT-PCR and found that *LFY*, *FY* and *AP1* were significantly downregulated in 20-day-old transgenic *Arabidopsis* (Figure S4G–I). These results indicated that the delayed flowering phenotype was associated with the suppressed expression of flowering-related genes in *PwNAC11* overexpression lines.

## 3. Discussion

### 3.1. PwNAC11 Positively Regulates Drought Resistance in Transgenic Arabidopsis

For most woody plants, it is still a great challenge to conducting functional verification in a homologous way via establishing transformation platforms [29]. Alternatively, we can still explore the gene function by heterogeneously transforming model plants. NAC transcription factors are reported to be involved in abiotic stress responses in various plant species, including model plants, crops and woody plants. Our previous work revealed that *PwNAC2*, cloned from *P. wilsonii*, could interact with PwRFCP1 to positively regulate abiotic stress response and enhance drought and salt resistance in transgenic *Arabidopsis* [28]. Another NAC, TF PwNAC30, acted as a negative regulator in plant responses to abiotic stress. Overexpression of *PwNAC30* apparently reduced the survival rates of transgenic *Arabidopsis* when subjected to drought and salt treatment [29], suggesting the distinct role and multiple function of different members of the NAC family from *P. wilsonii* in response to abiotic stress. Here, based on the high-throughput RNA-seq of the expression profiles of *P. wilsonii* in the absence or presence of drought treatments [30], we identified PwNAC11 as one of the most differentially expressed genes (DEGs) on a transcriptional level, which is a homologue of *Arabidopsis* NAC transcription factors ANAC019 and ANAC072 (RD26). Protein multiple sequence alignment and BLAST analysis showed that the conserved N-terminal of PwNAC11 protein had a typical NAM domain and its C-terminal was comparatively variable. Both the full length and C-terminal regions of PwNAC11 have transcriptional activation activity (Figure 1). Subcellular localization demonstrated that PwNAC11 was a nuclear-located transcriptional activator, which was consistent with most NAC transcription factors [33]. The transcript level of *PwNAC11* displayed a great increase at 3 h when *P. wilsonii* seedlings were exposed to drought stress, indicating its potential role in early response to abiotic stress. Furthermore, overexpression of *PwNAC11* in *Arabidopsis* considerably improved drought stress tolerance both for seedlings and adult plants. Under PEG treatment, the growth of the tested seedlings was inhibited to some extent, but the OE lines showed significantly higher germination rates, stronger vitality and longer root length compared with the WT and VC lines. Moreover, the survival rates of the OE lines were more than 60% higher than the WT and VC groups at the adult stage after dehydration followed by re-watering. Additionally, the RWC of the adult OE plants was nearly 20% higher than the control group (Figure 3). These results demonstrated that PwNAC11 functions as a positive regulator in response to drought stress throughout plants’ growth and development stages.

It is well known that drought can cause the accumulation of large amounts of ROS (mainly H_2_O_2_ and O2^−^) in chloroplasts and mitochondria [34]. The excessive accumulation of ROS can cause serious peroxidation damage to plant cell membranes. Therefore, ROS eliminating efficiency is an essential indicator of plant resistance to drought stress. In our study, the NBT and DAB staining for H_2_O_2_ and O_2_^−^ assays proved that ROS was slightly accumulated in the OE lines but accumulated extensively in WT and VC lines (Figure 4). Further measurement of enzymatic activities indicated that the activity of CAT, POD and SOD were upregulated in the OE lines compared to the lesser accumulation of MDA. These results indicated that PwNAC11 inhibits membrane lipid peroxidation via activating the ROS-scavenging system and decreasing the accumulation of ROS in resistance to drought stress.

Chlorophyll fluorescence is another important parameter reflecting the function of Photosystem II (PSII) and the transfer efficiency of electrons from PSII to PSI [35]. The Fv/Fm ratio and ΦPSII value represent the potential maximum light energy conversion efficiency and actual quantum yield of PSII, respectively [36,37]. However, the delicate photosynthetic system is frequently affected by the external environment, especially drought stress, which can decrease the Fv/Fm ratio and ΦPSII value. In our study, despite these indicators of different samples showing a declining trend under drought conditions, the *PwNAC11* OE lines displayed higher Fv/Fm, ΦPSII and chlorophyll content compared with the WT and VC groups. Additionally, significantly, the values of ΦPSII in transgenic lines remained almost unchanged whether in the presence or absence of drought treatment, suggesting the minimizing damage to the photosynthesis apparatus of PSII in *PwNAC11* OE lines and *PwNAC11* can stabilize photosynthetic energy utilization under drought conditions. Meanwhile, the NPQ, an indicator of plant to disperse excess light energy in the form of heat to protect itself under adverse conditions, showed no significant differences under normal and dehydration conditions in control groups, while *PwNAC11* OE lines showed increased NPQ values after drought treatment. These results verified the complete network of photoprotective mechanism in OE lines [38]. Recent studies revealed that the inhibition of light energy conversion could produce more excess excitation energy and promote the accumulation of ROS in plant [39]. In grape, Sun et al. found that the inhibition of Alternative oxidase (AOX) pathway increased the accumulation of H_2_O_2_, thus resulting in the photoinhibition under heat conditions [40]. Overexpression of Arabidopsis *AtCBF3* enabled transgenic potatoes to possess a favorable ROS removing ability, which decreased the degree of photoinhibition and enhanced heat resistance [41]. Therefore, we conclude that the efficient ROS-scavenging system in *PwNAC11* OE lines may partly contribute to the stable light energy conversion efficiency under drought conditions.

Many NAC TFs involved in stress response were reported to have an effect on plant development or flowering. For example, EsNAC1, an NAC TF isolated from *E. salsugineum*, was proven as a positive regulator of salt and oxidative stress but inhibited the process of vegetative growth [42]. Additionally, MLNAC5, a stress-related transcription factor from *Miscanthus*, improved drought and cold tolerance in transgenic *Arabidopsis* but inhibited the growth of the plant and accelerated the process of leaf senescence [43]. The overexpression of sugarcane NAC transcription factor *ScNAC23* in *Arabidopsis* accelerated flowering compared with WT plants [44]. Our previous study also revealed that another *P. wilsonii* transcription factor, PwNAC2, enhances drought and salt tolerance in plants and can interact with PwRFCP1 to co-regulate flowering via regulating the expression of *SOC1*, *FLC* and *FT* [28]. These results indicate that the different NAC transcription factors function differentially, and some of them can balance and coordinate the regulation of plant growth and flowering and coping with adverse stress. In this study, a late flowering phenotype was discovered in 35S: *PwNAC11* OE lines. Further investigation demonstrated that PwNAC11 delayed flowering time by inhibiting the expression of genes such as *LYF*, *FT* and *AP1*. Interestingly, the growth retardation disappeared when the plants were subjected to drought conditions (Figure 3A). Previous research showed that under nonstress conditions the growth inhibition phenotype was partly affected by the constitutive expression of 35S promoter [45]. Under normal conditions, for example, ectopic expression of *35S: AtDREB1A* in *Salvia miltiorrhiza* inhibited plant growth but the overexpression of stress-inducible *RD29A*: *AtDREB1A* caused no growth suppression. Therefore, we cannot exclude the effect of 35S promoter on flowering time in *PwNAC11* transgenic *Arabidopsis*. To further determine whether the stunted growth phenotype was mainly caused by functional *PwNAC11* expression or 35S promoter, knock-out studies are required to verify this hypothesis and decipher the role of PwNAC11 on flowering regulation in *P. wilsonii* in the future. In addition, considering most transcription factors are simultaneously involved in plant growth, development and response to stress, the stress-induced promoters or self-gene promoters are probably recommended for plant gene transformation to reduce the impact on the development process of the plant itself.

### 3.2. PwNAC11 Improves Drought Tolerance in an ABA-Dependent Manner

Plants’ responses to drought stress are usually controlled by hormonal signals, in which ABA acts as a mediator to regulate a range of processes in stress-signaling pathways [46]. CmBBX19, a zinc finger protein, suppressed drought resistance in chrysanthemum in an ABA-dependent way [47]. At a translational level, alternative splicing of rice NAC transcription factor ONAC054 led to premature termination of translation in response to exogenous ABA, thus producing more ABA-responsive ONAC054β variants to positively modulate the homeostasis [48]. In our study, expression of *PwNAC11* was greatly upregulated especially at 3 h after ABA treatment. *PwNAC11* overexpression lines conferred hypersensitivity to ABA at an earlier phase than the WT and VC groups. Meanwhile, some ABA synthesis genes, such as *NCED3* and *ABI5*, showed transcriptional abundance after exposure to PEG. These results implied that PwNAC11 is likely to participate in water-deficit regulation in an ABA-dependent pathway. In addition, previous investigations revealed that most TFs were involved in the regulation of stomata closure under the control of ABA signals. The heterologous expression of *AINAC1* in *Arabidopsis* increased the endogenous ABA level, thus resulting in an enhanced percentage of close stomata and reduced water loss [49]. CsMYB61, a MYB TF from *Citrus sinensis*, was implicated in the process of stomatal closure. Overexpression of *CsMYB61* driven by a stomata-specific promoter conferred a repressed opening of stomata pores, especially under the treatment of exogenous ABA [50]. Similarly, *AtMYB61* transgenic *Arabidopsis* displayed a higher percentage of closed stomata under drought treatment [51]. Here, we found that PwNAC11 acted in a similar way to regulate ABA-induced stomatal closure. Overexpression of *PwNAC11* transcript in *Arabidopsis* led to a significant increase in the number of close stomata (Figure 6). It was reported that stomatal closure is generally triggered by drought-induced hormone ABA as well as the application of exogenous ABA [52,53]. Our results also demonstrate that PwNAC11 functions as an ABA signal factor by promoting stomatal closure and ABA synthesis to directly or indirectly improve plants’ tolerance to drought stress.

The ABF TFs are considered as pivotal recipients of ABA signaling and function downstream of various ABA-mediated stress response, especially drought and salt stresses [54,55]. *ABI5*, an important ABA synthesis gene, is governed by AtABF3 in *Arabidopsis*, which could enhance salt stress tolerance by activating the ABA signal pathway [56]. In *Populus euphratica*, PeABF3 could activate the expression of *PeADF5* to enhance drought tolerance and promote ABA-induced stomatal closure [57]. Although different ABFs exhibited high levels of homology, they could also work antagonistically in definite conditions to generate a negative feedback effect. Pear ABF protein PpyABF3 can activate *PpyDAM3* expression by binding to the ABRE motif on *PpyDAM3* promoter, while the binding affinity of PpyABF3 to *PpyDAM3* promoter was disturbed by interacting with PpyABF2, thus revealing the antagonistic regulatory network of ABFs towards downstream genes [58]. In the present study, we found that PwNAC11 could physically interact with AtABF3 by Y2H and BiFC assay, which is consistent with another homologous gene, *ANAC072*, in *Arabidopsis*. ANAC072 participated in the ABA-responsive signaling pathway by interacting with ABF3 [59]. The data showed the conservation of function and further proves that NAC TFs could perform similar functions in both woody plants and *Arabidopsis*. However, ANAC072 was reported to promote leaf senescence and accelerate chlorophyll degradation, which was not observed in *PwNAC11* transgenic lines.

### 3.3. The Regulatory Networks of ABA-Induced Drought Resistance Mediated by PwNAC11

TF acts as a transcriptional activator/repressor by specifically binding to cis-acting elements in the promoters of target genes. Accordingly, it will enable us to uncover the unknown regulatory mechanisms by identifying the relative target genes. *ERD1* encoded a homologous protein of ATP binding subunit in *Escherichia coli* [13], and the expression of *ERD1* was much upregulated by abiotic stress or dark-induced senescence. In *Arabidopsis*, a zinc finger homeodomain TF (ZFHD1) could enhance the expression of *ERD1* by binding to its promoter region, which contributed to an obvious improvement of drought stress endurance in the *ZFHD1* overexpression lines [60]. Likewise, we found that PwNAC11 bound to the promoter of *ERD1* and activated *ERD1* expression. RT-qPCR analysis also showed that the expression of *PwNAC11* reached its peak at 3 h after PEG treatment, while at 6 h for *ERD1*. We next performed a Y1H assay to prove that PwNAC11 could specifically bind to ABRE motifs instead of DRE motifs. These results provide genetic evidence that PwNAC11 functions as a transcriptional activator upstream of *ERD1*, leading to enhanced dehydration resistance in an ABA-dependent manner. In addition, our results also confirmed the interaction between PwNAC11 and ABF3 (Figure 7), both of which could bind to the ABRE elements (Figure 8), but whether PwNAC11 and ABF3 simultaneously bind to the same ABRE element on ERD1 promoter, and whether the interaction of PwNAC11 and ABF3 interferes with the binding affinity to target genes remains to be further investigated. Nevertheless, the expression of PwNAC11 promoted the activation of the reporter gene by 2.67-fold greater than empty vector in tobacco, while ABF3 limitedly activated LUC expression. However, the simultaneous expression of PwNAC11 and ABF3 together enhanced the activation of the *ERD1* promoter more than PwNAC11 alone (Figure 8D). These results showed that PwNAC11 positively regulates plant response to early drought stress and the interaction of PwNAC11 and ABF3 further improves this activation by binding to the ABRE motifs. Generally, ABF3 can interact physically with diverse components to promote or inhibit the expression of downstream targets, especially stress-responsive genes. For example, in *Arabidopsis*, *AtSOC1* was ubiquitously expressed in different vegetative tissues and accelerated flowering. *At*ABF3 could form a complex with NF-Y TF and activated the expression of *AtSOC1*, and this phenotype was totally abolished in *nf-yc3 yc4 yc9* mutants [61]. Similarly, the physical interaction between CmBBX19 and CmABF3 in *Chrysanthemum* withheld the activation of *CmRAB18*, resulting in decreased tolerance to drought stress [47].

However, our results by no means suggest the minimal role of ABA-independent pathway present in PwNAC11-mediated drought stress response, since DRE-BINDING PROTEIN 2A (DREB2A) also physically interacted with PwNAC11 by Y2H and BiFC assay in this study (Figure 7). It was noteworthy that dehydration-responsive element (DRE) also exists in the promoter of *ERD1*, which is recognized as an essential cis-element for ABA-independent regulation in response to dehydration and heat stresses [62]. In woody plants, the expression of *BpDREB2*, a transcription factor of DREB protein family in *Broussonetia papyrifera*, was remarkably increased under salt and dehydration conditions, but no obvious change was observed under ABA treatment. Importantly, overexpression of *BpDREB2* in *Arabidopsis* improved its salt and freezing tolerance [63]. Similar results were found in *Jatropha curcas*. The expression patterns of *JcDREB* showed that it was induced by cold, drought and salt stresses, not by ABA signal [64]. However, some reports have also suggested that DREB transcription factors can be mediated in both ABA-dependent and ABA-independent pathways in response to abiotic stress. In *Leymus chinensis*, the transcript of *LcDREB3a* accumulated in response to ABA treatments and the protein was shown to bind to DRE motifs, indicating that *LcDREB3a* was involved in both ABA-dependent and ABA-independent signal transduction in the process of abiotic stress response. Here, we also found that PwNAC11 remarkably improved the transcription activity of *ERD1* promoter by interacting with DREB2A rather than directly binding to the DRE motif. We thus speculate that the drought response of *ERD1* is also modulated in an indirect ABA-independent approach, in which PwNAC11 may also participate. This assertion is also supported by the apparent up-regulation of *DREB2A* in transgenic *Arabidopsis*, whose expression levels are less dependent on ABA signals [65,66].

**Figure 9:**
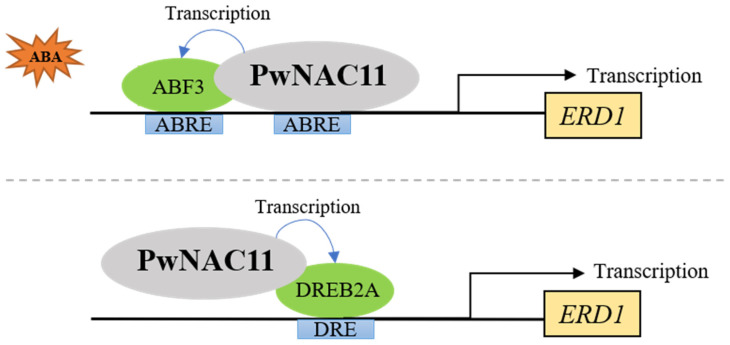
Proposed model of ABA-dependent and ABA-independent abiotic stress responses via PwNAC11.

In addition, combined with the data in our study that PwNAC11 was dramatically induced at 3 h after PEG treatment, while ABF3 highly expressed at 12 h and DREB2A at 6 h, we speculated that PwNAC11 plays a dominant role in positively regulating plants’ responses to early drought stress, and ABF3 or DREB2A were subsequently induced and synergistically regulate the *ERD1* expression. Based on these results and the function of homologous genes, we sketched the regulatory mechanism and linkage of ABA signals and the drought response pathway mediated by PwNAC11 (Figure 9). First, PwNAC11 functions to improve the expression of ABF3 or DREB2A at transcriptional-level control under drought stress, then the protein interaction between PwNAC11 and ABF3 strengthens the transcription of target genes by activating ABRE-containing promoters or indirectly activating DRE-containing promoters by interaction with DREB2A. The existence of such synergistic pathways could help plants to respond to environmental stress efficiently and rapidly, but it is necessary to further explore how precise networks PwNAC11 participate or contribute to the drought stress response via ABA-dependent or -independent pathways, which will provide further information on the drought tolerance mechanism of *Picea wilsonii*.

## 4. Materials and Methods

### 4.1. Plant Materials and Gene Expression Analysis

For RT-qPCR assay, the 4-week-old *P. wilsonii* seedlings grown on nutrient soil were watered with 20% polyethylene glycol (PEG) or treated with 100 µM ABA solution and the 2-week-old *Arabidopsis* seedlings were watered with 10% PEG solution. The whole plants were then harvested at 0, 3, 6 and 12 h after treatment for RNA extraction. Total RNA was extracted from *P. wilsonii* or *Arabidopsis* with plant RNA extraction kit (Kangwei Century Biotechnology Co., Ltd., Beijing China) and first strand cDNA synthesis kit (ABM, Beijing, China), followed by RT-qPCR analysis. Step One plus Real-time PCR system (ABI, Vernon, CA, USA) was used to perform RT-qPCR reactions with SYBR Green Master Mix enzymes (ABI, Vernon, CA, USA). The 2−ΔΔCT method was employed to calculate expression levels and all experiments were repeated 3 times for biological replications and 3 times for technical replications. *PwEF-1α* in *P. wilsonii* and *ACTIN* in *Arabidopsis* were selected as housekeeping genes in qRT-PCR assay, respectively.

Before sowing in the MS medium, all seeds were sterilized by 5% NaClO solution for 20 min. After 3 days dark treatment, the seeds were then placed in a 21 °C incubator under a 16-h light/8-h dark photoperiod at 70% relative humidity. At 14-days-old, plants were transferred into soil.

### 4.2. Bioinformatic Analysis

The sequences of PwNAC11 homologous proteins were obtained from NCBI database (https://www.ncbi.nlm.nih.gov/ (accessed on 1st November 2018)). Multiple sequence alignments of 12 NAC amino acid sequences were analyzed using Clustal X 1.83. The neighbor-joining method was subsequently used to construct the phylogenetic tree via MEGA-X (Mega Limited, Auckland, New Zealand) program.

### 4.3. Plant Phenotype Experiments under Drought and ABA Treatments

Seeds of *Arabidopsis* have Col-0 genetic background. To generate transgenic plants, full length of *PwNAC11* CDS was cloned into pCAMBIA1205 vector, which was driven by 35S promotor [28]. The agrobacterium stains GV3101 containing PwNAC11-pCAMBIA1205 were subsequently transformed into *Arabidopsis* using floral dipping. Stable homozygous lines were selected by hygromycin resistance and all T3 seedings could survive on selective medium, in which two dependent lines named OE-2 and OE-3, with relatively higher expression levels, were chosen as experimental samples.

The WT and VC lines were set as controls and OE-2 and OE-3 lines were chosen for phenotype analysis. For the seed germination experiment, the disinfected seeds were sown on MS solid medium (100 seeds were sown for each line) containing various concentration of ABA (0, 0.5, 1.0 µM) and mannitol (0, 100, 200, 300 mM), respectively. After placing in a refrigerator at 4 °C for 3 days, the mediums were then transferred into an incubator as described above. The germination number was recorded once a day and the germination criterion was the exposure of radicle. For the root length experiment, the seeds of each line were sown in MS medium for germination and then transferred to MS medium supplemented with different concentrations of mannitol and ABA. For seedlings treated with drought stress, all of the lines were sown on MS medium for 2 weeks and then transferred to soil under normal conditions for another 14 days. For drought stress, the 4-week-old seedlings were dehydrated for 14 days and then re-watered for 3 days. Phenotypic differences were observed after 14 days of drought treatment and re-watering. Each experiment was repeated three times.

### 4.4. Subcellular Localization Assay

The CDS of *PwNAC11* was constructed on pCAMBIA1205 vector, which was controlled by CaMV35S promotor. The RACK1A-RFP plasmid, a nuclear-located marker [29], and recombinant vector were transfected into *Agrobacterium tumefaciens* GV3101 cells, respectively, and then co-infiltrated into *Nicotiana benthamiana* leaves, which were then placed in the incubator at 25 °C (day)/23 °C (night) for 48–72 h. The TCS SP8 fluorescence microscope (Leica, Wetzlar, Germany) was used to detect GFP fluorescence. Three infiltrated leaves from two *N. benthamiana* were regarded as three biological repeats for each experiment.

### 4.5. Trans-Acting Activity and YEAST Two-Hybrid (Y2H) Assay

The complete *PwNAC11* CDS and truncated N-terminal (pGBKT7-PwNAC11-1-153) or C-terminal (pGBKT7-PwNAC11-171-318) sequences of *PwNAC31* were cloned into pGBKT7 vector, respectively. The assay was performed as previously described with modification [29]. Briefly, the plasmids were separately transformed into yeast AH109 cells with empty pGBKT7 vector as negative control. The yeast strains were firstly painted on SD/-Trp medium and then transformed into SD/-Trp-His-Ade+X-α-gal selective medium.

For Y2H assay, similar transcriptional activity assays were conducted. The CDS of *ABF3*, *ZFHD1*, *RHA2A* or *ANAC019* were cloned to pGBKT7 vector as the prey and recombinant vectors were then selected on SD/-Trp-His-Ade+X-α-gal medium. Full-length CDS of PwNAC11 was correspondingly constructed on pGADT7 vector as the bait. The recombinant AD and BD vectors were simultaneously inserted into yeast AH109 cells and selected on SD/-Trp-Ura-His-Ade+X-α-gal medium.

### 4.6. Yeast One-Hybrid (Y1H) Assay

Y1H assay was accomplished with the Matchmaker Gold Yeast One-Hybrid System Kit (TaKaRa, Beijing, China). Briefly, about 2 kb *ERD1* promoter region and short tandem repeats containing core ABRE (ACGTG) and DRE (GTCGGC) motifs were constructed on pAbAi vector. The pGADT7-PwNAC11 or pGADT7-ABF3 vectors were transformed into competent cells of yeast containing the *ERD1* pro sequences. The recombinant vectors were selected on SD/-Ura-Leu+ AbA medium.

### 4.7. Dual-Luciferase Assay

Dual-luciferase assay was performed as previously described [67]. Briefly, the *ERD1* promoter was cloned into pGreenII 0800-LUC and the CDS of PwNAC11, ABF3 or DREB2A was cloned into pGreenII 62-SK vector. The transfected *Nicotiana benthamiana* leaves were detected by Dual Luciferase Reporter Gene Assay Kit (Beyotime, Shanghai, China). The firefly luciferase (fLUC) and renilla luciferase (rLUC) were detected by Multiscan Spectrum (Inginite M Plex, TECAN). The empty pGreenII 0800-LUC vector was used as control and each experiment was repeated three times.

### 4.8. Bimolecular Fluorescence Complementation Assay (BiFC)

The complete CDS of *PwNAC11* was cloned into pSPYCE vector to generate PwNAC11-cYFP fusion protein and coding region of *ABF3* was similarly cloned into pSPYNE vector. The recombinant vectors were transformed into agrobacterium strains GV3101 and then co-infiltrated into *Nicotiana benthamiana* leaves as mentioned above. The GFP signal was observed through Leica TCS SP8 fluorescence microscope.

### 4.9. Physiological Measurement

The Diaminobenzidine (DAB) and Nitro Blue Tetrazolium (NBT) staining assays were conducted as previously described [68]. NBT Chromogen and Metal Enhanced DAB Substrate Kit were employed in this experiment (Solarbio, Beijing, China). Briefly, the leaves of different lines were immersed in the staining solution for 2 h in darkness. Then, the staining solution was replaced by 95% alcohol and the leaves were boiled until decolorization. The catalase, antioxidant enzymes’ activities and proline content were measured by CAT, SOD, POD and Pro detection kits (Jiancheng Bioengineering Institute, Nanjing, China). The chlorophyll content was quantified by spectrometer as previously described [69]. The chlorophyll and fluorescence parameters of *Arabidopsis* leaves under drought stress were measured by handy portable fluorometer (PAM-2500, WALZ, Germany), and the fluorescence parameters were calculated as previously described [35]. Some parameters were calculated as follows: NPQ = Fm/Fm’ − 1, Fv/Fm = (Fm − Fo)/Fm, ΦPSII = (Fm’ − F)/Fm’, in which Fo is the minimum recorded fluorescence intensity in the dark-adapted states. Fm and Fm’ are the maximal recorded fluorescence intensity in the dark and light-adapted states, respectively.

### 4.10. Stomatal Aperture Measurements

Stomatal apertures were measured as described previously [70]. Leaves of *Arabidopsis* were pre-incubated in MES-KCl buffer for 2.5 h under normal light conditions, followed by MES-KCl buffer treatment alone or with 10 μM ABA for another 3 h. Stomatal pores of OE, WT and VC lines were photographed by light microscope DM2500 (Leica, Wetzlar, Germany).

### 4.11. Statistical Analysis

SPSS software version 18.0 (SPSS Corp, Chicago, IL, USA) was used for statistical analysis. Duncan’s multiple range test was performed for statistical difference analysis with *p* < 0.05 designated as significant difference.

## Data Availability

No new data were created or analyzed in this study. Data sharing is not applicable to this article.

## Supplementary Materials

Supplementary Materials can be found at https://www.mdpi.com/article/10.3390/ijms22136952/s1.
