# Supplementary material for: NAC Transcription Factor PwNAC11 Activates ERD1 by Interaction with ABF3 and DREB2A to Enhance Drought Tolerance in Transgenic Arabidopsis
Source: Int J Mol Sci. 2021 Jun 28;22(13):6952. doi: 10.3390/ijms22136952 (PMC8269012; doi:10.3390/ijms22136952)
Supplement: Supplementary file 1 [file ijms-22-06952-s001.zip › ijms-1261446-supplementary.pdf]

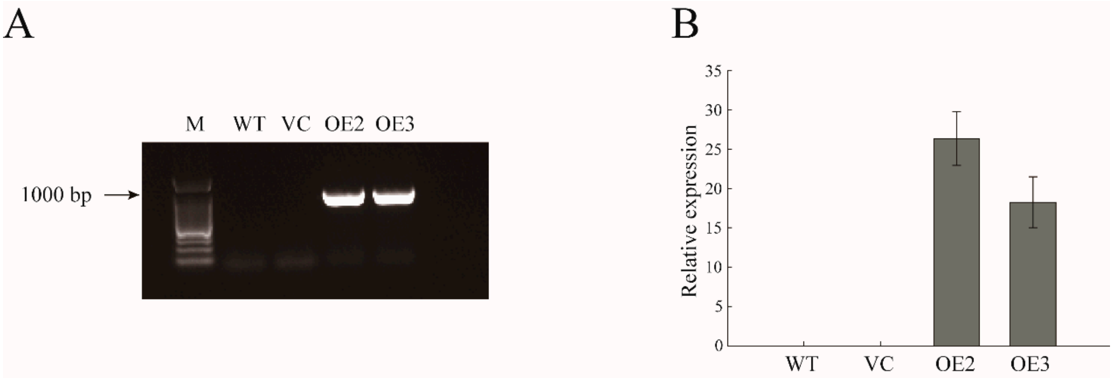

Figure S1. (A) The expression of PwNAC11 in transgenic *Arabidopsis* by RT-PCR. (B) The expression of PwNAC11 in transgenic *Arabidopsis* by RT-qPCR. Different letters indicate significant differences at *p*-value < 0.05.

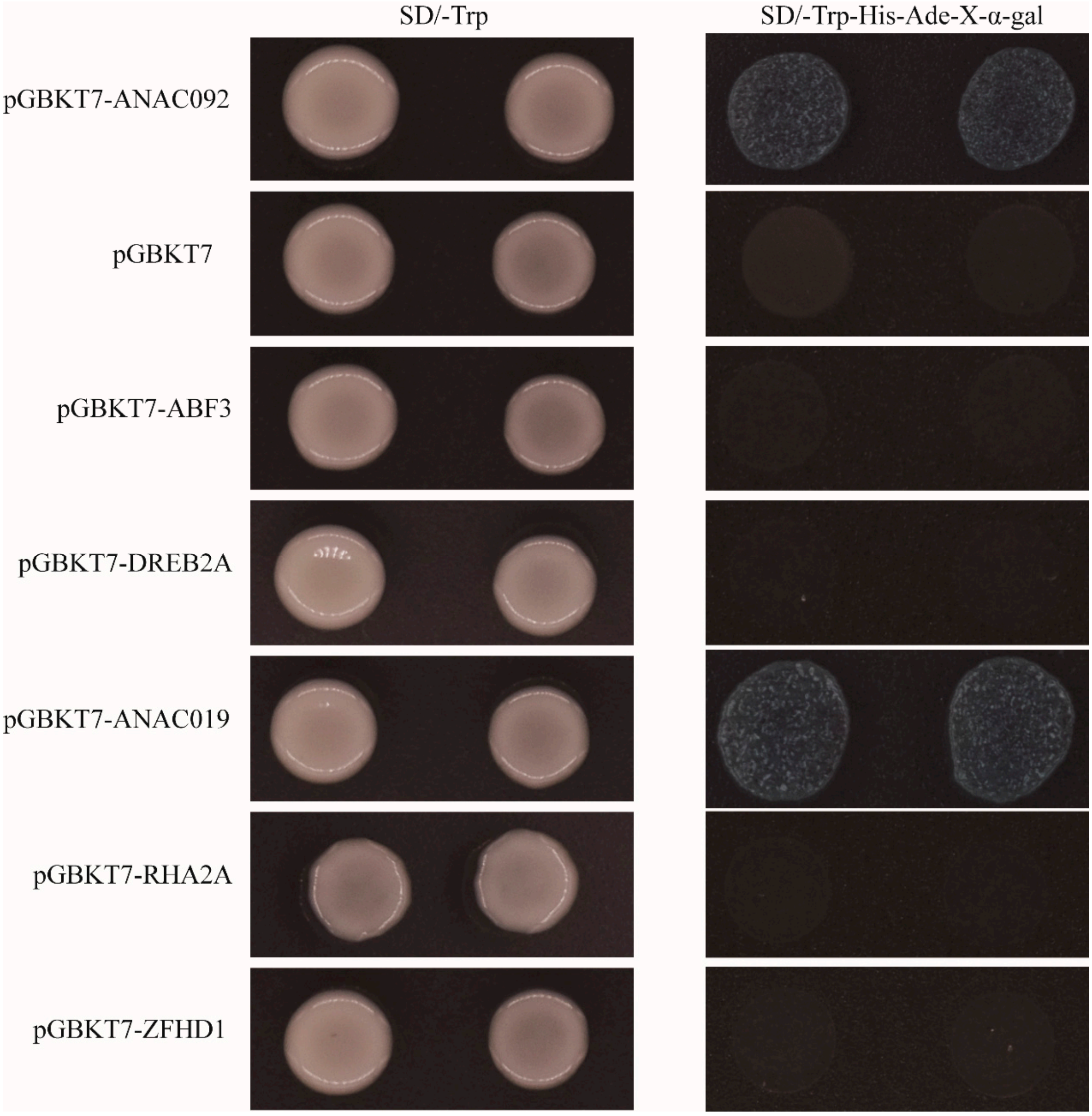

Figure S2. Yeast trans-acting activity assay for interacting proteins. pBD-ANAC092 was used as positive control.

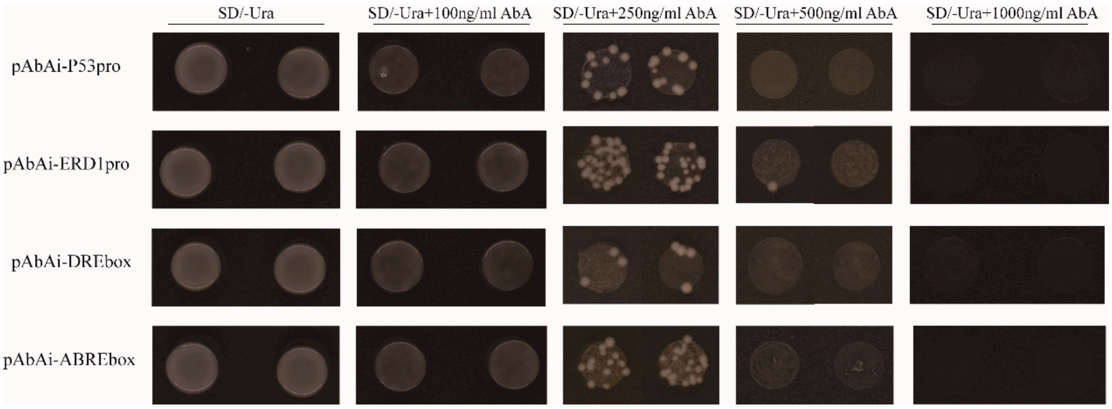

Figure S3. Screening of AbA concentration for yeast one-hybrid assays.

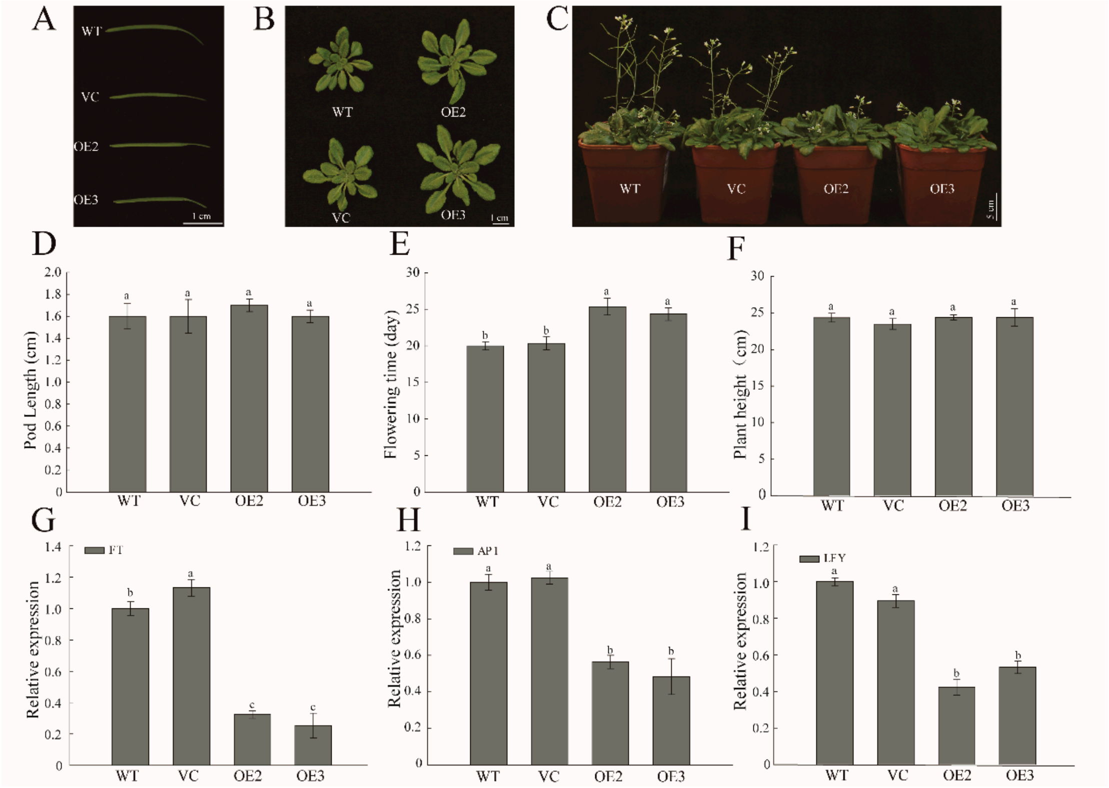

Figure S4. Effects of *PwNAC11* overexpression on plant growth and development. (A) Phenotypic assay of fruit pods in different lines under normal conditions. (B) Phenotypic assay of rosettes in each line under normal conditions. (D-F) Measurements of pod length, flowering time and plant height under normal conditions. (G-I) Expression patterns of some flowering-related genes. Different letters indicate significant differences at p-value < 0.05.

Supplementary Table S1. q-PCR primers used in this study

Gene Name	Gene No.		Sequences (5′ to 3′)
PwNAC11		Forward	AGCAGAAGAATAGCCAGACTGT
		Reverse	CAGGGCTCAGATTCATGGCA

PwEF-1α		Forward	AACTGGAGAAGGAACCCAAG
		Reverse	AACGACCCAATGGAGGATAC
ABF3	AT4G34000	Forward	GATGTGGTTAACCGTTCTCAAC
		Reverse	CAGCTTGCAGTAGATTGTTGTT
ATHB-7	AT2G46680	Forward	GCAAGTGGCTATATGGTTTCAG
		Reverse	GAAGCCAAGTTGTCGTAGTTTT
ZFHD1	AT1G69600	Forward	CTTCCGAGAATCTCAACTTCCT
		Reverse	TAAGAGCAAGGAACAGGAGAAG
NCED3	AT3G14440	Forward	GATGAATTTGTTCCAGAGAGCG
		Reverse	AACACTAGGATCAGCCGTTTTA
DREB2A	AT5G05410	Forward	CATGTTTGATGTCGATGAGCTT
		Reverse	ATTCCGTAGTTGAGGCTTTGTA
ANAC019	AT1G52890	Forward	AATTCAGCAACAACGGTACTTC
		Reverse	GGTTTTCTGTTCGGTTAAGTCC
ANAC055	AT3G15500	Forward	ACGAAGAGCTGATGGTTGAATA
		Reverse	TCCGTCGAGATAACTTTATCCG
ANAC032	AT1G77450	Forward	GATTGGACGATTGGGTATTGTG
		Reverse	AACGGAAAATCAAGGGCATTAC
ERD1	AT5G51070	Forward	CTTTCTCTATCAGCACGAAACG
		Reverse	CGGTGCGATATATTGACAATCC
FT	AT1G65480	Forward	CTACAACTGGAACAACCTTTGG
		Reverse	TGACAATTGTAGAAAACTGCGG
AP1	AT1G69120	Forward	CATTCTCGAAAAGAAGAGCTGG
		Reverse	ACTCCATCGACCAGTTTGTATT
LYF	AT5G11730	Forward	TAAAATGCGACACTACGTTCAC
		Reverse	CAGCTTTGTTGGAACATACCAA
ABI5	AT2G36270	Forward	AATAAGAGAGGGATAGCGAACG
		Reverse	GCTACCACCACCTCTATGTATC
ACTIN		Forward	GGTAACATTGTGCTCAGTGGTGG
		Reverse	AACGACCTTAATCTTCATGCTGC

Supplementary Table S2. Primers for vectors' construction in this study

Gene Name		Sequences (5′ to 3′)
ERD1pro-KpnI-pAbAi-F	Forward	TTCGAGCTCGGTACCAATTTGAATAAAGAAGAG
ERD1pro-SalI-pAbAi-R	Reverse	TGCCTCGAGGTCGACAGGAAGTAGATAACACCT
PwNAC11-NdeI-AD-F	Forward	GATTACGCTCATATGATGACACAGAAGGGT
PwNAC11-EcoRI-AD-R	Reverse	ACCCGGGTGGAATTCCTAATAAGACCTCTG
DREB2A-AD-NdeI-F	Forward	GATTACGCTCATATGATGGCAGTTTATGAT
DREB2A-AD-EcoRI-R	Reverse	ACCCGGGTGGAATTCTTAGTTCTCCAGATC
ABF3-AD-NdeI-F	Forward	GATTACGCTCATATGATGGGGTCTAGATTA
ABF3-AD-EcoRI-R	Reverse	ACCCGGGTGGAATTCCTACCAGGGACCCGT
p0800-*ERD1*-KpnI-F	Forward	GGCGAATTGGGTACCAATTTGAATAAAGAAGAG
p0800-*ERD1*-SalI-R	Reverse	ATCGATACCGTCGACAGGAAGTAGATAACACCT
SK-*PwNAC11*-BamHI-F	Forward	AGAACTAGTGGATCCATGACACAGAAGGGT
SK-*PwNAC11*-SalI-R	Reverse	CCCCTCGAGGTCGACCTAATAAGACCTCTG
SK-*DREB2A*-BamHI-F	Forward	AGAACTAGTGGATCCATGGCAGTTTATGAT
SK-*DREB2A*-SalI-R	Reverse	CCCCTCGAGGTCGACTTAGTTCTCCAGATC
SK-*ABF3*-BamHI-F	Forward	AGAACTAGTGGATCCATGGGGTCTAGATTA
SK-*ABF3*-SalI-R	Reverse	CCCCTCGAGGTCGACCTACCAGGGACCCGT
PwNAC11-AD-NdeI-F	Forward	GATTACGCTCATATGATGACACAGAAGGGT
PwNAC11-AD-EcoRI-R	Reverse	ACCCGGGTGGAATTCCTAATAAGACCTCTG
DREB2A-BD-NdeI-F	Forward	GAGGACCTGCATATGATGGCAGTTTATGATCAG
DREB2A-BD-SalI-R	Reverse	CCGCTGCAGGTCGACTTAGTTCTCCAGATC
ABF3-NdeI-BD-F	Forward	GAGGACCTGCATATGATGGGGTCTAGATTAAAC
ABF3-SalI-BD-R	Reverse	CCGCTGCAGGTCGACCTACCAGGGACCCGTC
RHA2A-NdeI-BD-F	Forward	GAGGACCTGCATATGATGGGGCTACAAGGT
RHA2A-SalI-BD-R	Reverse	CCGCTGCAGGTCGACTCAGTGGAGAGAGAA
ZFHD-NdeI-BD-F	Forward	TAAAATGCGACACTACGTTCAC
ZFHD-SalI-BD-R	Reverse	CAGCTTTGTTGGAACATACCAA
ANAC019-NdeI-BD-F	Forward	GAGGACCTGCATATGATGGGTATCCAAGAA
ANAC019-SalI-BD-R	Reverse	CCGCTGCAGGTCGACTCACATAAACCCAAA
